# Updates on traditional methods for combating malaria and emerging *Wolbachia*-based interventions

**DOI:** 10.3389/fcimb.2024.1330475

**Published:** 2024-04-23

**Authors:** Iqra Mushtaq, Muhammad Sajjad Sarwar, Ammara Chaudhry, Syed Ali Haider Shah, Muhammad Mubeen Ahmad

**Affiliations:** Department of Zoology, Faculty of Life Sciences, University of Okara, Okara, Pakistan

**Keywords:** *Anopheles*, malaria control, *Wolbachia*, insecticide resistance, drug resistance, integrated vector management (IVM), traditional control strategies

## Abstract

The escalating challenge of malaria control necessitates innovative approaches that extend beyond traditional control strategies. This review explores the incorporation of traditional vector control techniques with emerging *Wolbachia*-based interventions. *Wolbachia*, a naturally occurring bacteria, offers a novel approach for combatting vector-borne diseases, including malaria, by reducing the mosquitoes’ ability to transmit these diseases. The study explores the rationale for this integration, presenting various case studies and pilot projects that have exhibited significant success. Employing a multi-dimensional approach that includes community mobilization, environmental modifications, and new biological methods, the paper posits that integrated efforts could mark a turning point in the struggle against malaria. Our findings indicate that incorporating *Wolbachia*-based strategies into existing vector management programs not only is feasible but also heightens the efficacy of malaria control initiatives in different countries especially in Pakistan. The paper concludes that continued research and international collaboration are imperative for translating these promising methods from the laboratory to the field, thereby offering a more sustainable and effective malaria control strategy.

## 1 Introduction

The global prevalence of mosquito-borne diseases has been on the rise, fueled by factors such as increased travel, urbanization, and the diminishing effectiveness of traditional control measures. Among these diseases, malaria stands out as a significant global health problem, particularly affecting tropical regions. The *Plasmodium* parasites are transmitted through the bites of infected female *Anopheles* mosquitoes. Surprisingly, *Plasmodium falciparum* alone accounts for nearly 99.7% of all reported malaria cases worldwide (Ale et al., 2022). In sub-Saharan Africa, the burden of *falciparum* malaria is predominantly spread by primary mosquito vectors including *Anopheles gambiae*, *An. coluzzii*, *An. funestus* and *An. arabiensis* (Suh et al., 2023).

Malaria remains one of the world’s most devastating infectious diseases, affecting over 100 countries and resulting in hundreds of thousands of deaths annually (Ashour and Othman, 2020; Yu et al., 2022). Between, 2000 and, 2023, there were approximately 2 billion reported cases of malaria worldwide, resulting in 11.7 million deaths (World Health Organization, 2022a). Over one billion people, particularly in underdeveloped regions like Southern Africa, face the risk of malaria, and Pakistan is no exception to this threat. Significantly, there has been a four-fold surge in reported malaria cases in Pakistan following the floods in August, 2022. The country has been dealing with a malaria epidemic, with suspected cases reaching 3.4 million between January and August, 2022—a notable rise from the 2.6 million cases reported during the same period in, 2021 (World Health Organization, 2022a). It is noteworthy to mention that the COVID-19 pandemic has also significantly affected malaria control programs (Zawawi et al., 2020), causing setbacks despite global health initiatives. The pandemic has disrupted healthcare services, leading to an increase in malaria cases. Resource redirection, overwhelmed healthcare systems, and reduced access to malaria interventions have contributed to this rise, highlighting the importance of maintaining essential services for disease control during health crises. In underdeveloped nations worldwide, controlling malaria remains challenging, even with traditional methods like bed nets and indoor spraying.

In many underdeveloped countries, traditional control methods like indoor residual spraying (Jeffries et al., 2018) with chemical insecticides such as DDT and the use of pyrethroid-treated nets have been relied upon for vector control (Unwin et al., 2023). However, these methods come with logistical and financial challenges and have contributed to the emergence of insecticide resistance in mosquitoes. The recent emergence of artemisinin-resistant strains of *Plasmodium* in Africa adds to the growing concerns (Skorokhod et al., 2023). This could be a warning sign for global efforts to control malaria. These challenges highlight the need for novel, effective, and sustainable approaches to malaria control.

The rise in insecticide resistance and the potential environmental consequences of chemical control methods have raised questions about the long-term viability of these strategies. Traditionally, the development of new antimalarial drugs has been a key component of the fight against malaria. However, the emergence of drug-resistant strains poses significant risks and challenges (Yu et al., 2022). Resistance has also emerged in mosquitoes to insecticides and in *Plasmodium* parasites to antimalarial drugs (Skorokhod et al., 2023). To address these challenges and the need for more effective and sustainable malaria control strategies, there is growing interest in innovative approaches like *Wolbachia*-based interventions (Walker and Moreira, 2011).

*Wolbachia* is an endosymbiotic bacterium that naturally occurs in many mosquito species but notably not in *Anopheles* mosquitoes, the primary vectors for malaria transmission (Hughes and Rasgon, 2014). This unique bacterium has the potential to disrupt mosquito-borne diseases through various mechanisms, including cytoplasmic incompatibility (CI), lifespan shortening, and pathogen interference (El-Shaarawi and Piegorsch, 2002; Fox et al., 2023). *Wolbachia*’s CI induction leads to an effective mosquito and disease control strategy. Through this and other mechanisms such as lifespan shortening and direct pathogen interference, *Wolbachia* presents unique biological control strategies for mosquito and disease prevention (Iturbe-Ormaetxe et al., 2011; Sarwar et al., 2022).

A virulent *Wolbachia* strain called *w*MelPop has been found to shorten the adult lifespan of its natural host, *Drosophila melanogaster*, despite the typically non-virulent associations that *Wolbachia* often forms with its hosts (Min and Benzer, 1997). The life-shortening effect of the *w*MelPop strain was seen as having potential applications in controlling mosquito-borne diseases. *Anopheles* mosquitoes are a suitable choice for *Wolbachia*-based control methods because they usually do not naturally harbor *Wolbachia* (Baldini et al., 2014). Transinfection of mosquitoes with maternally inherited *Wolbachia* strains is one of the most effective methods (Cook and McGraw, 2010; Iturbe-Ormaetxe et al., 2011; Bourtzis et al., 2014). *Wolbachia* boosts arthropod resistance against viruses and reduces their reproductive abilities.

**Figure 1:**
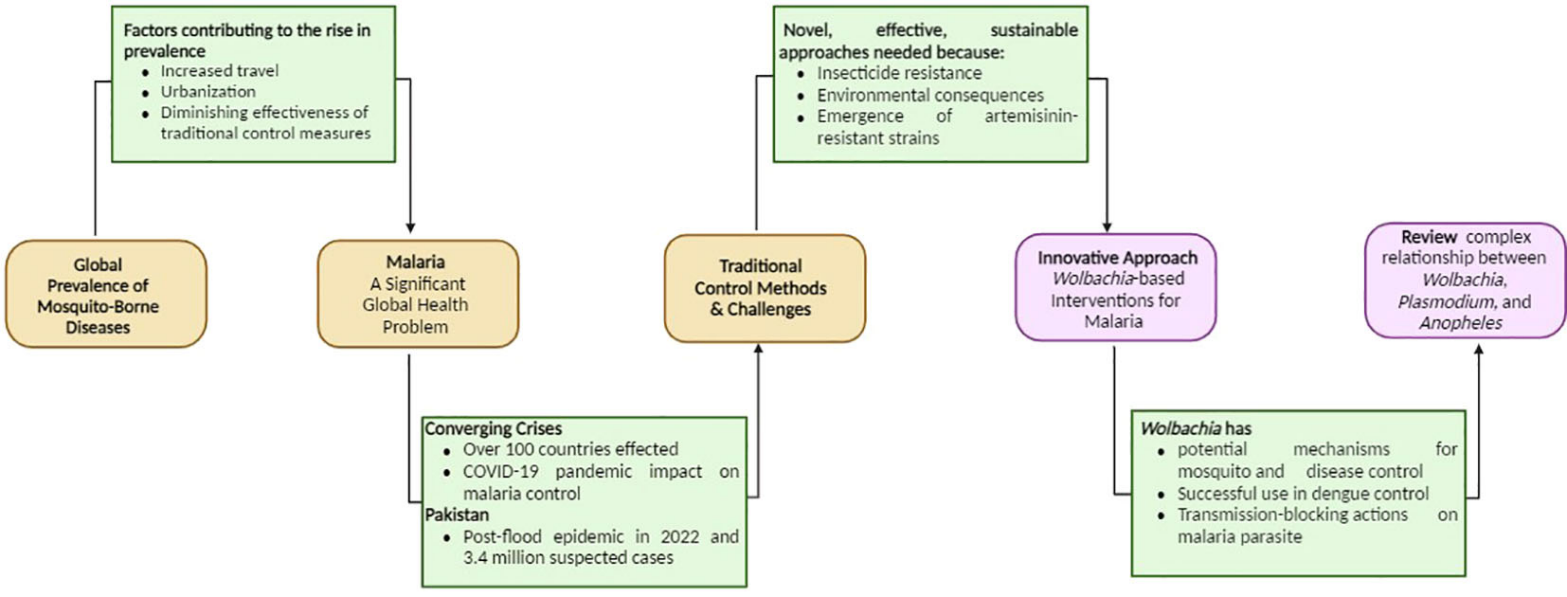
A schematic diagram of introduction.

*Wolbachia* has shown promise in controlling dengue virus transmission, gaining recognition and support from the WHO and health authorities. This approach involves the broader concept of employing biological interventions to combat vector-borne diseases. While *Wolbachia* primarily targets arboviruses in mosquitoes, similar innovative strategies could be explored for malaria control, including genetically modified mosquitoes, insecticides, or biological agents to disrupt the malaria parasite’s life cycle. The success of *Wolbachia* in dengue control highlights the potential for creative approaches in addressing other mosquito-borne diseases like malaria. *Wolbachia* has some transmission-blocking actions on the malaria parasite. It has been shown to have a powerful anti-sporozoite action and could greatly lower the amount of sporozoites in the mosquito stage (Gomes et al., 2017). This review article aims to not only analyze the drawbacks of current control strategies but also explore the complex relationship between *Wolbachia*, *Plasmodium*, and *Anopheles* mosquitoes as an innovative approach to malaria control (**Figure 1**).

## 2 Vector control methods

Malaria transmission has seen a decrease in past decades, primarily through interventions targeting mosquito vectors, like bed nets and indoor residual sprays (Jeffries et al., 2018). Yet, malaria persists in much of sub-Saharan Africa due to challenges, including insecticide-resistant vectors, limited intervention coverage, outdoor vector biting, and transmission by secondary vector species, which compromise the effectiveness of these control measures. The challenges in malaria control are multifaceted and concerning. A consistent shortfall in funding for malaria control efforts (Cohen et al., 2012), the emergence of mosquito resistance to pyrethroids, the primary insecticide used in bed nets, and other classes of insecticides for indoor residual spraying, and the global spread of parasite resistance to artemisinin, poses a severe threat to malaria control effectiveness. In addressing these challenges, an effective method is larval source management (LSM), focusing on mosquito larvae. Malaria control can target adult mosquitoes to reduce biting and survival, or immature mosquitoes to lower overall numbers. Long-lasting insecticide-treated nets (LLITNs) and IRS (Jeffries et al., 2018) focus on adults, while LSM prevents immature mosquitoes from becoming adults.

### 2.1 Insecticide resistance in malaria vectors

The extended incubation period of *Plasmodium* in *Anopheles* mosquitoes necessitates the predominant use of insecticide interventions, such as IRS and insecticide-treated nets (ITNs), to effectively lower vector survival rates and control malaria transmission (Enayati and Hemingway, 2010). In malaria-prone areas, these tools play a crucial role in control efforts and can even lead to local elimination in certain cases. But in high-transmission regions like parts of sub-Saharan Africa, where individuals may face up to 1,000 infectious mosquito bites annually (Hay et al., 2000), existing interventions can only reduce annual inoculation rates by approximately one-tenth. In regions with exceptionally high transmission rates and in areas where the main disease-carrying mosquitoes are resistant to these control methods, additional interventions are necessary (Shaukat et al., 2010).

Insecticide resistance in *Anopheles* mosquitoes, reported since the, 1950s, now threatens malaria control, risking a disease resurgence (Suh et al., 2023). Insecticide resistance is a global concern, affecting over 500 insect species, including over 50 *Anopheles* species responsible for transmitting malaria to humans (Manikandan et al., 2022). Large-scale efforts, including ITNs, indoor spraying, and improved case management, reduced malaria cases from 81.1 per, 1000 in, 2000 to 58.9 in, 2015 (Suh et al., 2023). However, progress slowed as insecticide-resistant mosquitoes spread in endemic areas. Progressive malaria burden reduction via improved insecticide-based vector control is threatened by the widespread emergence of chemical resistance. This resistance is primarily genetic, resulting from the selection of specific genetic modifications through migration and mutation. The application of insecticides, both in agriculture and public health, has been a significant factor in driving resistance in malaria vectors. Supported by four mechanisms (metabolic, target site, cuticular, and behavioral resistance), this phenomenon involves changes in resistant vectors that can affect *Plasmodium* parasite development. While molecular diagnostics have been developed to detect target-site resistance, metabolic resistance is complex but has seen recent progress in identifying key detoxification enzymes (Odero et al., 2023). Additionally, other physiological and behavioral changes in mosquito populations contribute to resistance, but their impact on insecticide efficacy remains poorly understood. Vector mosquitoes have developed resistance to major chemical classes used in public health, including pyrethroids, DDT, carbamates, and organophosphates. Despite this resistance, pyrethroid insecticides are increasingly used in IRS and LLITNs (World Health Organization, 2021). Naturally, the use and switching of these pesticides have led to the development of resistance.

LLITNs are effective at preventing indoor biting and resting by malaria vectors, but their effectiveness diminishes when people are not in bed, especially during early mornings or outdoor activities in the evening (Monroe et al., 2020). This means that engaging in outdoor activities, such as farming and security work, along with certain cultural practices, can heighten the risk of malaria transmission. There are challenges faced by malaria control programs, including inadequate financial support, limited access to LLITNs, and poor attitudes of residents towards ownership and usage of LLITNs. Environmental Health Officers face challenges such as political interference, lack of educational materials for health education and promotion activities, lack of means of transportation, and recognition by government authorities (Agyemang-Badu et al., 2023).

The continuous use of LLITNs and the ongoing interaction between LLITNs and malaria vectors have led to significant threats, including resistance to the insecticides used for impregnation and the emergence of behavioral avoidance strategies among malaria vectors. For instance, *An. arabiensis* has adapted to avoid LLITNs by biting and resting outdoors and feeding on cattle rather than humans (Meza et al., 2019; Kreppel et al., 2020), while *An. funestus*, highly anthropophilic and resistant to pyrethroids (Meza et al., 2019), has altered its biting times to avoid LLITN interventions and maintain malaria transmission during drier periods. Meza et al. (2022) have documented the resistance and behavioral avoidance of *An. arabiensis* and *An. funestus* to LLITNs insecticides, but the extent of their ability to bypass LLITNs and feed on their designated hosts is yet to be confirmed. *An. funestus* has demonstrated a notable capacity to penetrate all types of nets compared to *An. arabiensis*. This ability, combined with other factors, represents an additional risk for ongoing malaria transmission across Africa (World Health Organization, 2022b).

Cross-resistance and multiple resistance pose significant challenges to achieving the Millennium Development Goals for malaria control. Therefore, ongoing surveillance and monitoring campaigns are crucial for Malaria Control Programs, to design more effective and sustainable strategies for malaria vector control at a practical level. Inefficiently combating malaria in all endemic areas, preserving and improving the efficacy of current insecticide-based therapies is the first challenge. To do this, new pesticides, particularly pyrethroids, must be developed to overcome resistance to existing pesticides.

**Figure 2:**
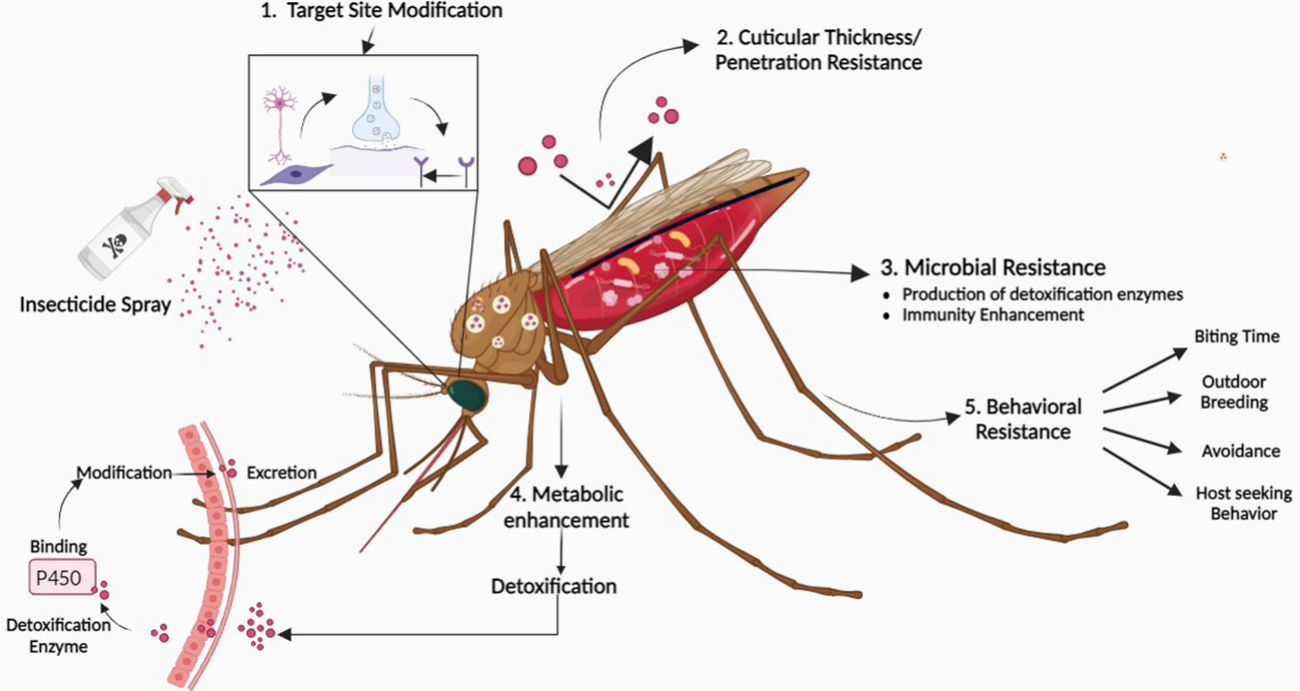
Evolution of resistance in mosquitoes against insecticides. 1. Target site modification occurs when the target site is altered, rendering it unable to interact with the insecticide 2. Cuticle thickening prevents insecticide entry by increasing thickness and reducing permeability 3. Detoxification enzymes and microbiota in mosquitoes detox insecticides by breaking down toxins into harmless compounds 4. Metabolic enzymes break down or modify insecticides before they can harm target sites 5. Behavioral resistance happens when insects change their behavior to avoid insecticide exposure. Figure created in BioRender.com.

We need strategies for using new insecticides effectively while limiting the emergence of resistance, along with rapid and cost-effective methods to detect significant pesticide resistance. This issue is significant since a lot of bed nets coated with pyrethroid pesticides are used in malaria control programs. However, mosquitoes are evolving pyrethroid resistance in regions like sub-Saharan Africa where malaria is pervasive (Ranson et al., 2009). The second problem is the development of interventions for *Anopheles* mosquito species, which include dozens of species with various behaviors that are not successfully targeted by present techniques. Different types of resistance in mosquitoes have shown in **Figure 2**.

Insecticide resistance in malaria vectors can have complex and multifaceted effects on vector biology, including longevity, which can impact the transmission of malaria. Understanding these interactions is essential for developing effective strategies for malaria control and prevention. Above discussed traditional vector control approaches like LLITNs and IRS have their limitations, particularly when it comes to long-term sustainability. The development of insecticide resistance in mosquitoes is a growing concern, and it jeopardizes the efficacy of such strategies (Ranson et al., 2009; Enayati and Hemingway, 2010; Nalinya et al., 2022). In addition, different species of *Anopheles* mosquitoes exhibit various behaviors that are not effectively targeted by current methods (Shaukat et al., 2010). In light of the decreasing effectiveness of mosquito control due to insect resistance, urgent innovation is needed to develop novel approaches.

### 2.2 Evolution of drug resistance

The challenges facing malaria control are complex and manifold. The increasing resistance of malaria parasites to antimalarial drugs has become a significant concern in recent times, drawing considerable attention from researchers and healthcare authorities. Four different types of intracellular protozoan parasites can infect humans with malaria. There are differences between *Plasmodium falciparum*, *P. vivax*, *P. ovale*, and *P. malariae* in terms of their geographic range, microscopic appearance, clinical characteristics, and possibility for the emergence of drug resistance. Drug resistance among different *Plasmodium* species, like *P. falciparum* and *P. vivax*, is a growing concern that has been making the treatment increasingly challenging. The search for antimalarial drugs has been particularly critical because drug resistance could negate the progress made so far in disease control. Recent research has shown that Africa is home to malaria strains that are resistant to the artemisinin drugs, posing a threat to the entire world (Lubell et al., 2014).

For malaria to be controlled, finding novel medications is still crucial; nevertheless, issues come from rising drug resistance. Antimalarial resistance first came out around the end of the, 1950s when Southeast Asia and South America started to demonstrate resistance to chloroquine. Chloroquine resistance in the, 1970s and, 1980s caused a resurgence of the disease, especially in Africa and the tropics (Baird, 2009). *P. vivax* was formerly thought to be quite harmless and to have negligible antimalarial resistance when compared to *P. falciparum*. Recent data, however, suggests that *P. vivax*-related severe illness is becoming more common. Chloroquine treatment failure in *P. vivax*-infected patients has also been documented.

Vaccination strategies have also been a focal point of research. Several vaccines are in development, targeting different stages of the parasite’s lifecycle. However, these vaccines often focus on a single antigen, and this specificity may limit their effectiveness given the diverse strains of malaria and the transient nature of immunity they confer. Multi-stage vaccines that target both the asexual and sexual stages of the parasite have shown promise but are not without challenges. There are three categories of malaria vaccines: Pre-erythrocytic vaccines (target sporozoites and liver stages of the parasite), blood-stage vaccines (address asexual blood stages of the parasite), and transmission-blocking vaccines (aim to disrupt sexual stages and mosquito midgut antigens to prevent further transmission of the disease) (Carter et al., 2000; Richie and Saul, 2002). The impact of these vaccines is also subject to other factors of a host, including the parasite’s genetic diversity and the host’s immune response.

The only medication that the WHO now recommends for preventing malaria transmission is primaquine (Rajasekhar et al., 2023). Due to safety concerns in individuals with glucose-6-phosphate dehydrogenase deficiency, it is not commonly utilized despite its ability to eliminate mature gametocytes from the blood (White et al., 2012). Developing vaccines is a lengthy process with numerous challenges, while certain biological control strategies offer solutions for malaria transmission blockage (Caragata et al., 2020). Transmission blocking of malaria could occur at different levels such as at the sexual stages of *Plasmodium* (Wang et al., 2017), transfer of infection from patient to vector (Yu et al., 2022), fertilization of gametocytes into zygotes, development of *plasmodium* in the midgut of mosquitoes, and transmission of *Plasmodium*.

Historically, malaria vaccines used a single antigen from various parasite stages. Multi-stage target vaccines addressing asexual and sexual stages have shown promise. For instance, a vaccine containing a fusion protein of *P. vivax* circumsporozoite and P25 proteins demonstrated significant protective and blocking effects (Mizutani et al., 2014). Similarly, certain compounds like benzimidazole derivatives and internal peroxy compounds have exhibited dual effects against both asexual and sexual stages of *Plasmodium* (Miranda et al., 2014). However, vaccines designed to target various stages of mosquito development did not exhibit synergistic effects. In one study, a transmission-blocking vaccine against pre-fertilization antigen Pys48/45 and post-fertilization antigen Pys25 had a stronger blocking effect than Pys48/45 alone but was weaker than Pys25-based vaccines (Zhu et al., 2017).

**Figure 3:**
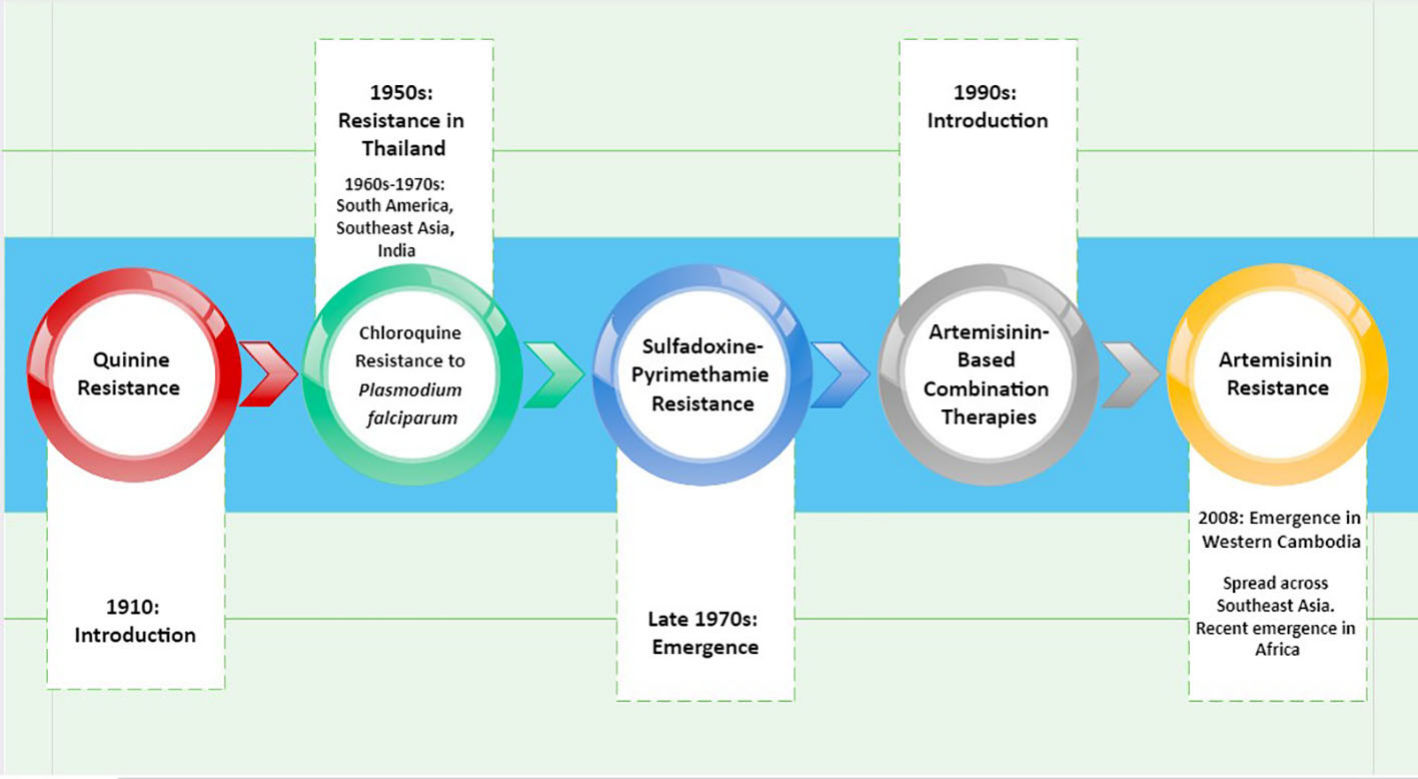
Evolution of resistance against antimalarial drugs.

Designing vaccines involves multiple resource-intensive, long-term steps, including target selection, production, protein folding, and adjuvant discovery. *Plasmodium* parasites have evolved resistance to antimalarial drugs, limiting the effectiveness of pharmacological interventions (**Figure 3**). For instance, artemisinin resistance has already been reported in Africa (Lubell et al., 2014). Although vaccines are being developed, they have yet to provide a comprehensive solution to the problem (Schuerman and Ockenhouse, 2022). While traditional methods for malaria control are making progress, their limitations necessitate the search for innovative alternatives. Studying genetic molecular markers in malaria provides insights into drug action and resistance mechanisms, crucial for improving treatment and transmission control.

### 2.3 Larval source management and complexity of *Anopheles*

Effective malaria control relies on collecting data about vector distribution and population density in the area to plan and implement precise control measures. *Anopheles* mosquito abundance varies significantly between houses within the same villages. *An. gambiae* is attracted to humans, while *An. arabiensis* prefers animals. Therefore, the mosquito population in a house is likely linked to the presence of humans and livestock (Minakawa et al., 2002). *An. gambiae* is typically prevalent in damp environments and *An. arabiensis* is more common in arid regions (Minakawa et al., 2002). *An. gambiae* breeds in diverse, man-made water bodies, including puddles, rice paddies, sunlit rivers, streams, quarries, and construction sites (Ndiaye et al., 2020). Unlike *An. gambiae*, *An. funestus* typically breeds in large, semi-permanent, or permanent water bodies with emergent vegetation. Despite being a significant malaria vector, *An. funestus* breeding sites are surprisingly hard to find, despite the size and permanence of their habitats (Nambunga et al., 2020).

Disease control programs are needed to identify the breeding sites of mosquitoes. Changes in land use, like agricultural expansion, can create new mosquito breeding sites for vectors that transmit malaria and other diseases. LSM reduces malaria vector densities by addressing mosquito breeding sites. It has substantially lowered malaria transmission in various settings (Asale et al., 2019; Dambach et al., 2019) and played a crucial role in malaria elimination in countries such as Brazil, Italy, the USA, Israel, Sri Lanka, and China (Hardy et al., 2023). Importantly, LSM targets outdoor-biting mosquitoes. However WHO advises using larviciding primarily in areas where mosquito breeding sites are few, fixed, and easily locatable, considering logistical challenges and resource constraints.

The development of novel larvicides and delivery methods has been notable, but the crucial area for enhancement lies in the accurate and timely mapping of breeding sites. The rapid advancement in mapping technologies holds the potential to revolutionize the way larval source management is conducted, making it more effective in targeting mosquito breeding sites. Traditional methods use ground surveys, but now Earth Observation (EO) data like drones and satellites are increasingly used to quickly identify potential breeding sites and direct control efforts (Hardy et al., 2017; Byrne et al., 2021). Infrastructure data, alongside ground-based vector surveys, is crucial for planning vector control interventions. To effectively detect *Anopheles* breeding sites, it’s essential to collect recent and temporally accurate EO data, as traditional satellite data may not sufficiently capture small, vegetated, or transient habitats due to infrequent collection and limitations like cloud cover (Carrasco-Escobar et al., 2022).

Drone technology has the power to change how we carry out LSM for malaria control. Drones offer the potential to transform malaria vector control by swiftly and precisely mapping potential mosquito larval habitats. This data is valuable for guiding field LSM strategies. Drones can capture highly detailed images of the ground below, with resolutions better than 10 centimeters, quickly and without high costs. This is especially crucial for malaria, where mosquito breeding sites can be tiny. As satellite-based methods can’t provide this level of detail, they make drones a valuable tool in the fight against malaria (Hardy et al., 2017). The Technology-Assisted Digitizing (TAD) mapping approach using the RegionGrow tool was found to be significantly more accurate in extracting information about potential mosquito larval habitats from drone imagery compared to the supervised classification approach. Integrating the TAD approach into operational LSM programs is recommended due to its high accuracy results (Hardy et al., 2022).

LSM is a vital tool for malaria control, endorsed by the WHO. It has been effective in global malaria elimination efforts. However, it is often labor-intensive due to challenges in identifying breeding sites. Drone imaging technology shows promise in mapping these sites, but questions about its operational use and cost still need answers (Hardy et al., 2023). However the accuracy, and cost-effectiveness of a drone and smartphone-based mapping system, in comparison to conventional larviciding and the standard of care need further investigation.

Vector control should be community-based. While eliminating mosquito larval habitats in individual households is essential, it alone cannot effectively reduce mosquito populations in the entire community. Although the prevalence of malaria has seen some decline during past years globally, it is still unknown which common preventative strategy is most successful in preventing malaria infection. According to Musoke, Atusingwize (Musoke et al., 2023) the use of multiple malaria prevention methods effectively reduced malaria incidence, prevalence, human biting, and mosquito inoculation rates, while increasing mosquito deterrence and mortality. However, some studies showed mixed results or no benefits of using multiple approaches to prevent malaria, indicating the need for more evidence on the effectiveness of certain prevention methods used individually or in combination (Wangdi et al., 2018). According to Agyemang-Badu et al. (2023), environmental management and sanitation can be a promising strategy for malaria vector control, particularly in areas with outdoor residual transmission. Adherence to building regulations to prevent encroachment of natural wetlands and revision of fines for sanitary offenders are important measures to support environmental management and sanitation. Close collaboration with provincial and national government officials is emphasized as a key factor for successful outcomes, suggesting the importance of maintaining and strengthening these partnerships in future efforts (Kollipara et al., 2023).

## 3 *Wolbachia*-based control

The mosquito stage is crucial for understanding disease transmission and developing strategies to block it. Additionally, it’s where genetic diversity is generated through sexual recombination, which can potentially promote the spread of drug resistance and pose challenges for effective vaccine development. Because of resistance caused by physiological and behavioral changes, common mosquito control measures such as chemical pesticides and environmental management are only somewhat successful. Non-target insects, especially pollinators, are also affected by chemical interventions. There is an urgent need for safe, long-term solutions to reduce the malaria burden. There has recently been an increase in the development of genetic control technologies that use transgenic mosquitos to achieve population suppression or population modification. Mosquitoes that are pathogen-resistant or refractory are designed to be released into wild populations so that their heritable modifications can propagate and prevent disease transmission.

Malaria is spread to people by infective female *Anopheles* mosquitoes while they are feeding on human blood. There are 475 officially classified species in the genus *Anopheles*, and there are about 70 vector species or species complexes that are important for public health (Jeffries et al., 2018). A variety of microbiota are encountered by *Plasmodium* parasites throughout the mosquito infection cycle, both in the midgut and other tissues of the insect. Numerous investigations have demonstrated that specific bacterial species can block *Plasmodium* development (Micks et al., 1961; PuMPUNI et al., 1993; Pumpuni et al., 1996; Straif et al., 1998; Gonzalez-Ceron et al., 2003).

**Figure 4:**
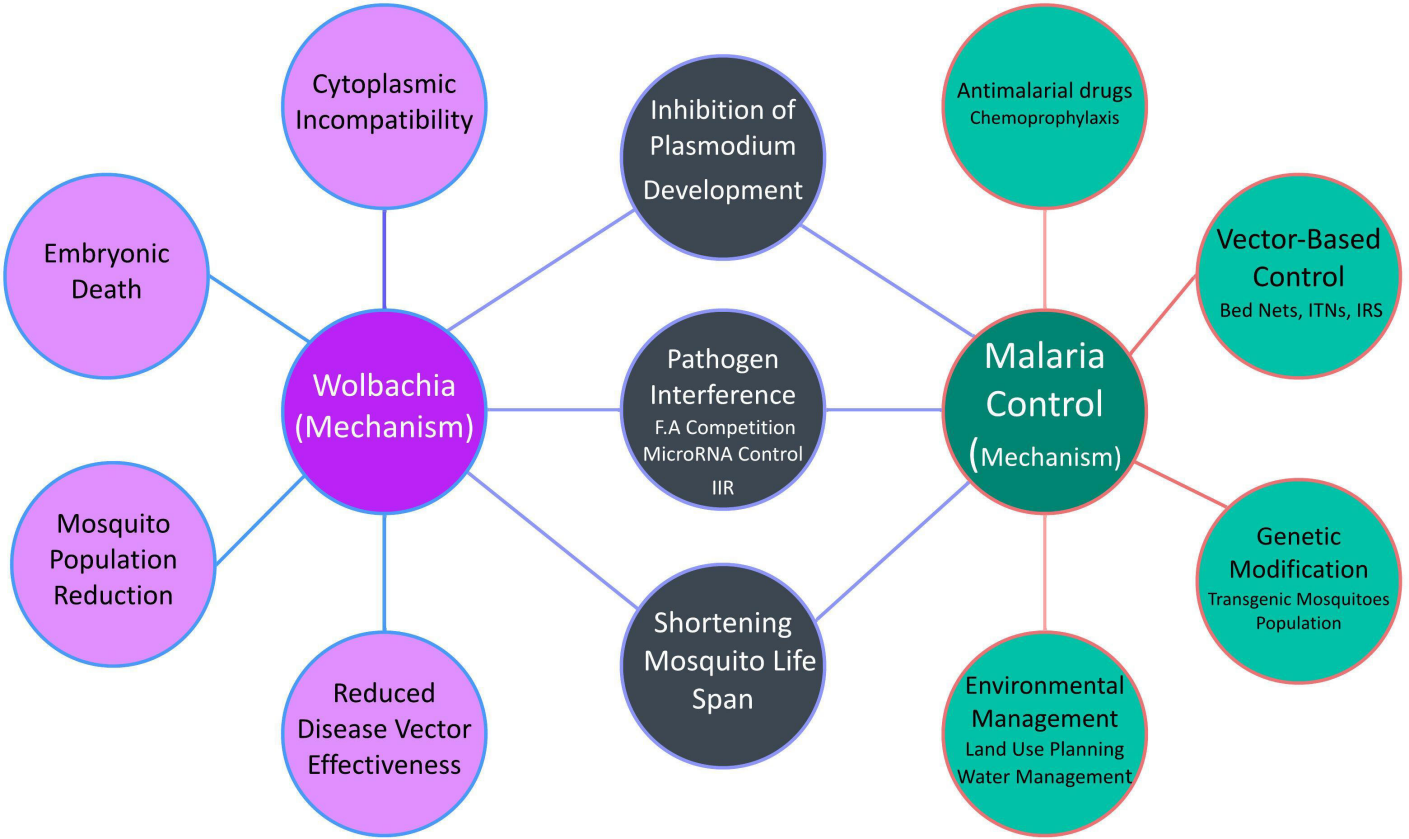
Integration of *Wolbachia* with Malaria control strategies.

*Wolbachia* has gained a lot of attention in recent years as a potential bio-replacement method in malaria management. *Wolbachia*-based control operates primarily through three mechanisms: cytoplasmic incompatibility, pathogen interference, and lifespan shortening of the host mosquito (Hoffmann et al., 2015). When *Wolbachia*-infected males mate with uninfected females, CI causes embryonic death, reducing the mosquito population (Sinkins, 2004). Pathogen interference involves the inhibition of *Plasmodium* development within the mosquito, thereby reducing the likelihood of malaria transmission (Moreira et al., 2009a). *Wolbachia* can shorten the lifespan of mosquitoes, making them less effective as disease vectors (Ogunlade et al., 2021). *Wolbachia’*s ability to prevent transmission during the *Plasmodium*’s mosquito stage of development is currently of much greater interest to researchers. Studies suggest that *Wolbachia* can inhibit pathogens through competition for fatty acids, host microRNA control, and enhancing innate immune responses (**Figure 4**) (Geoghegan et al., 2017).

### 3.1 Discovery of *Wolbachia* strains in *Anopheles*

*Wolbachia* is not often carried by all mosquitoes, but infections can be experimentally transferred to other mosquito species to produce host strains with beneficial traits for disease control. It’s interesting to note that significant vector genera, including *Anopheles*, were previously thought to be *Wolbachia*-free, but *Wolbachia* was recently found in *An. gambiae* in West Africa (Baldini et al., 2014). Several studies have recovered *Wolbachia* DNA from several native anopheline populations throughout the last decade. Natural *Wolbachia* infections in malaria vectors disprove prior theories that anopheline vectors are fundamentally resistant to these endosymbionts. The co-existence of other bacteria, particularly those from the genus Asaia (Hughes et al., 2014a), was one hypothesized explanation for *Wolbachia* absence in *Anopheles* species. This acetic acid bacterium is consistently linked to several *Anopheles* species and frequently predominates in the microbiota of mosquitoes. In laboratory studies, Asaia was found to prevent the vertical transmission of *Wolbachia* in *Anopheles*, and it was also discovered to have a negative relationship with *Wolbachia* in mosquito reproductive tissues.

If a specific species lacks *Wolbachia*, a *Wolbachia* strain must be injected to produce a *Wolbachia*-infected colony to use for control. The relationship between the donor and recipient hosts influences the result of *Wolbachia* transinfection. Asian malaria vector *An. stephensi* is the only one that has achieved stable transinfection to date (Bian et al., 2013). Walker, Quek (Walker et al., 2021) reveal novel *Wolbachia* strains discovered in *Anopheles* mosquitoes. *An. moucheti*, a significant vector in forest areas of equatorial Africa, has recently been found to have a severe *Wolbachia* infection in its ovaries. Renewed optimism has been offered in extending this biocontrol method to important African malaria vector species by the additional discoveries that these natural *Wolbachia* infections were passed to offspring by their mothers and that they can produce the CI phenotype.

Most research on the prevalence of *Wolbachia* in *Anopheles* species has been limited to Asia and up until, 2014, there has been no indication of strains in this genus of mosquitoes (Jeffries et al., 2018). *Wolbachia’s* presence in wild *Anopheles gambiae* mosquitoes was initially shown in Burkina Faso (Baldini et al., 2014). Hughes et al. (2014b) demonstrated that a stable maternally transmitted *Wolbachia* population can be established in *An. gambiae* and *An. stephensi* by using antibiotics to suppress other insect microbiota.

**Figure 5:**
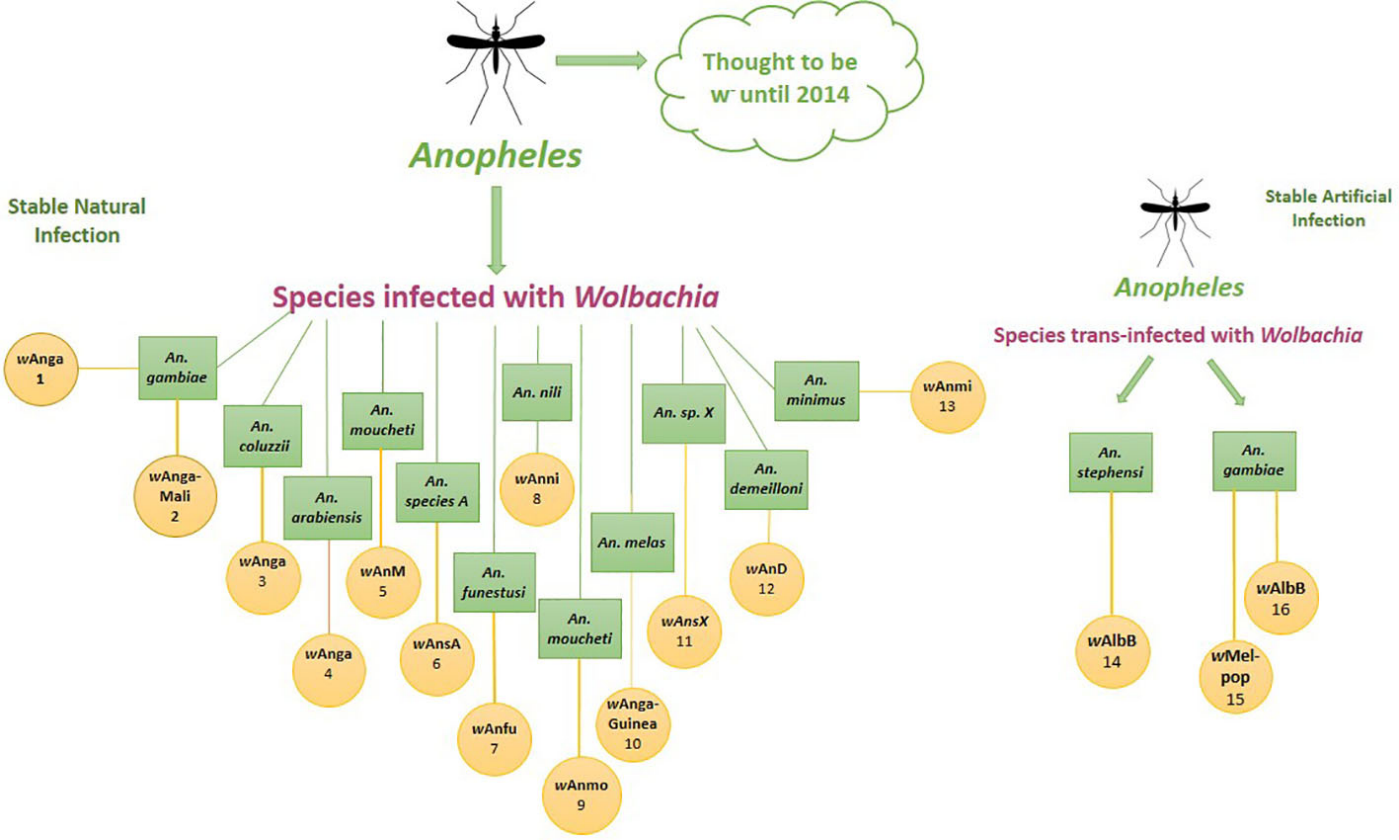
*Anopheles* mosquito hosts and associated *Wolbachia* strains. 1. (Baldini et al., 2014), 2. (Gomes et al., 2017), 3. (Shaw et al., 2016) 4. (Baldini et al., 2018) 5. (Jeffries et al., 2018) 6. (Jeffries et al., 2018) 7. (Niang et al., 2018) 8. (Ayala et al., 2019), 9. (Ayala et al., 2019), 10. (Jeffries et al., 2021), 11. (Jeffries et al., 2021) 12. (Walker et al., 2021) 13. (Walker et al., 2021). 14. (Bian et l., 2013), 15. (Hughes et al., 2011).

In the meantime, there is mounting proof that *Wolbachia* infections are now spreading in mosquitoes (Jeffries et al., 2021) and 25 species of African *Anopheles* mosquitoes have been discovered to carry 16 different types of *Wolbachia* infections (Ayala et al., 2019). Recent findings have identified native *Wolbachia* strains (collectively referred to as *w*Anga) in the *An. gambiae.* The *w*Anga strain was found to effectively infect reproductive tissues and somatic tissues where *Plasmodium* development occurs, potentially competing for resources and boosting the immune response against malaria parasites. Similar results were observed with the *w*Anga-Mali strain in Mali (Gomes et al., 2017). Tongkrajang et al., 2020 (Tongkrajang et al., 2020), reported that *An. minimus* harbor *w*Anmi. *w*Ang was also discovered in *An. arabiensis* by Bldini et al. (Baldini et al., 2018). Molecular phylogeny analysis, using the 16S rRNA gene, has identified the presence of at least two distinct *Wolbachia* genotypes in *An. fenustus*. These genotypes have been designated as *w*Anfu-A and *w*Anfu-B, indicating that they represent different genetic variants or strains of *Wolbachia* within the host organism (Niang et al., 2018). This genetic diversity among *Wolbachia* strains is common and can have various implications for host biology and interactions. This discovery suggests that *Wolbachia* may be widespread within the *An. gambiae* complex across Sub-Saharan Africa, potentially impacting malaria transmission. Moreover, globally, there are around 70 *Anopheles* vector species capable of transmitting malaria, and recent molecular barcoding studies have unveiled a greater diversity of these species than morphological identification alone, suggesting an underestimated diversity of malaria vectors with possible resident *Wolbachia* strains (**Figure 5**) (Jeffries et al., 2018).

### 3.2 Effect of *Wolbachia* on *Plasmodium*

The lifespan of the mosquito has a substantial impact on parasite growth and can affect malaria transmission. *Plasmodium’s* extrinsic incubation period in its hematophageal host lasts between 11 and 14 days. As a result, only mosquitos with long enough lifespans could allow the malaria parasite to fully mature to the sporozoite infective stage and participate in disease transmission. Bacteria found in wild *Anopheles* populations can influence malaria vector competence, and the mosquito microbiome can influence mosquito immunology. In addition to its effects on the mosquito, *Wolbachia* can impede the malaria parasite, potentially lowering the amount of sporozoites in the mosquito stage and preventing transmission (Gomes et al., 2017). Using embryonic microinjection, Joshi, Pan (Joshi et al., 2017) introduced the *w*AlbB strain from *Ae. albopictus* into *An. stephensi*, established the first maternally inheritable *Wolbachia* infection in an *Anopheles* malaria vector, resulting in the transinfected LB1 strain in, 2011. *w*AlbB causes the LB1 mosquito to become resistant to the human malaria parasite *P. falciparum* and a stable *wMelPop* infection in *Aedes aegypti* also has refractory effects on *P. gallinaceum*. A transitory *wMelPop* infection consistently reduced both *P. falciparum* and *P. berghei* in *An. gambiae* (Kambris et al., 2010; Hughes et al., 2011). *P. falciparum* suppression has similarly been observed in *An. gambiae* with a transitory *w*AlbB infection. Additionally, the observed reduction in malaria transmission was linked to the increased expression of anti-Plasmodium immune genes, such as Thioester-containing protein 1 (*TEP1*) (Kambris et al., 2010). It was stated that native *w*Pip infection might worsen avian malaria *P. relictum* parasite in the *Culex pipiens* mosquito (Zele et al., 2014). The rodent parasites *P. berghei* and *P. yoelii* oocyst loads in the midgut of *An. gamibiae* and *An. stephensi*, respectively, were both reported to rise during a temporary *w*AlbB infection. *Wolbachia* strains *w*MelPop and *w*AlbB infections in *An. gambiae* have been demonstrated to significantly suppress *P. falciparum* (Hughes et al., 2011). In Burkina Faso, a study by Shaw et al. (2016) revealed that *Wolbachia* infections in natural *Anopheles* populations influence egg-laying. These infections have a negative correlation with *Plasmodium* development in the mosquitoes. In, 2020, Wong et al. (2020) reported that *Wolbachia* infection in *An. gambiae* has two significant effects: It reduces the mosquito’s lifespan and provides resistance to pathogen infections in these mosquitoes **Table 1**.

**Table 1:** Transfected strains of *Wolbachia* in *Anopheles* and their effects on *Plasmodium*.

Strain	Host	Parasite	Effect on parasite infection	Source
***w*AlbB**	*An. gambiae*	*P. berghei*	Rise	(Hughes et al., 2012)
***w*AlbB**	*An. gambiae*	*P. falciparum*	Suppress	(Hughes et al., 2014b)
***w*AlbB**	*An. stephensi*	*P. berghei*	Suppress	(Joshi et al., 2017)
***w*AlbB**	*An. gamibiae*	*P. berghei*	Rise	(Zele et al., 2014)
***w*AlbB**	*An. stephensi*	*P. yoelii*	Rise	(Zele et al., 2014)
***w*MelPop**	*An. gambiae*	*P. berghei*	Suppress	(Hughes et al., 2012)
***w*MelPop**	*An. gambiae*	*P. falciparum*	Suppress	(Hughes et al., 2011)
***w*Anga**	*An. gambiae*	*P. falciparum*	Suppress	(Gomes et al., 2017)
***w*Anga**	*An. coluzzii*	*P. falciparum*	Suppress	(Gomes et al., 2017)

### 3.3 How does *Wolbachia* control *Plasmodium*?

Anti-*Plasmodium* immunity has been demonstrated in many studies. According to research, *Wolbachia* caused *An. stephensi* mosquitoes to express anti-*Plasmodium* immune genes such as *TEP1*, *LRIM1*, Toll pathway gene *Rel1*, and the effector Defensin (Joshi et al., 2017). The insect’s innate immune response includes various defense mechanisms such as epithelial barriers, and local and systemic immune reactions. The cellular immune response in insects is carried out by hemocytes and involves mechanisms like phagocytosis, encapsulation, coagulation, and melanization. These mechanisms generate Reactive Oxygen Species (ROS) at infection sites to eliminate pathogens. It has been observed that some species produce ROS as a defense against bacterial or fungal infection. It’s interesting to note that *An. gambiae* needs high enough ROS levels to establish a successful immune response against bacteria and *Plasmodium*. Mitochondria in mosquito midgut cells can produce increased ROS levels to combat *Plasmodium* (Gonçalves et al., 2012). Insects’ systemic immune response involves the production of antimicrobial peptides (Agyemang-Badu et al., 2023) by the fat body, which is then released into the hemolymph. AMP gene expression is primarily regulated by two signaling pathways: the Imd pathway, activated in response to Gram-negative bacteria, and the Toll pathway, triggered by Gram-positive bacteria, fungi, and yeast (Buchon et al., 2014).

Insect immunity, involving the production of AMPs and ROS regulated by the Toll and Imd pathways, effectively combats a wide range of pathogens, including Gram-positive and Gram-negative bacteria, fungi, yeast, and protozoa like *Plasmodium*. *Wolbachia* is a gram-negative bacteria and global expansion of *Wolbachia* is aided by mutualistic interactions like better host fertility and longevity or protection against infections (Zug and Hammerstein, 2015). *Wolbachia*’s contrasting immune responses in native and novel hosts are intriguing. In their native hosts, they evade AMP-based immunity (Moreira et al., 2009a). However, when introduced into new hosts, their strong induction of AMP gene expression likely involves the Imd pathway, which responds to DAP-type peptidoglycan (PG) from Gram-negative bacteria (Vollmer et al., 2013). Despite *Wolbachia* lacking a conventional cell wall and PG detection being unconfirmed, they may possess the ability to synthesize DAP or a similar molecule.

*Wolbachia* induces an antipathogenic effect in hosts, primarily enhancing resistance and tolerance to pathogens, particularly viruses, with a stronger antiviral effect observed. The strength of this effect correlates positively with *Wolbachia* density. The insect immune system plays a pivotal role, as when *Wolbachia* is introduced into uninfected hosts or those infected with different strains, it triggers upregulation of host immune genes, especially those in the Toll and Imd pathways. This immune response leads to the production of antimicrobial peptides, which is thought to underlie the observed antipathogenic effects, particularly the protection against viruses in mosquitoes (Moreira et al., 2009a). Upregulation of immune genes in the Toll/Imd pathways cannot universally explain *Wolbachia*-induced antipathogenic effects, as shown in various studies in both native and novel hosts. There is also some potential function of ROS in *Wolbachia*-induced antipathogenic actions.

**Figure 6:**
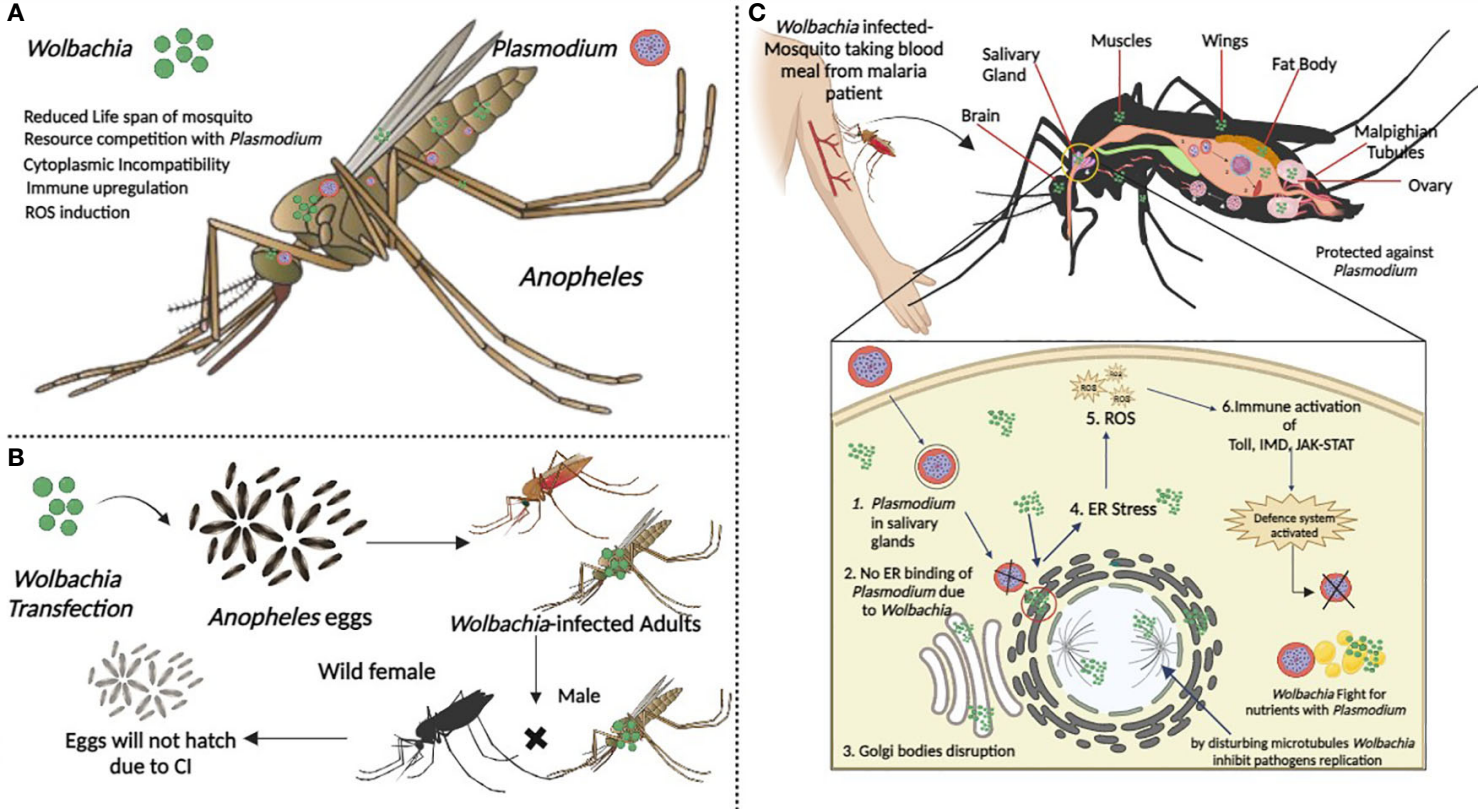
*Wolbachia*, *Plasmodium* and *Anopheles*. **(A)** Co-occurrence of *Wolbachia* and *Plasmodium* in *Anopheles* mosquito and possible effects of *Wolbachia* on host **(B)** When *Wolbachia* infected male mate with uninfected or wild female its results in CI. **(C)** *Wolbachia* in different body parts of mosquito. *Plasmodium* stages in *Anopheles*. Fusion of male and female gametes 2. Formation of zygote 3. Formation of ookinete 4. Formation of oocyst 5. Release of sporozoites from mature oocyst 6. Sporozoites enter into salivary gland. In detailed view of how *Wolbchia* prevents *Plasmodium* from replication in salivary glands. Figure created in BioRender.com.

The mechanism of *Wolbachia*-mediated pathogen interference has been the subject of two major theories: either *Wolbachia* infection primes the host’s immune system to fight off other pathogens, or *Wolbachia* and pathogens compete for metabolic resources like cholesterol (**Figure 6**) (Pujhari et al., 2023). Sheth (2023) revealed the upregulation of immunity genes, including TEPs and complement-like system components, in both midguts and carcasses upon *Wolbachia* infection. TEPs (*TEP1*, *TEP3*, and *TEP14*) have their impact on *P. falciparum* infection. TEP1 may play a role in *An. stephensi*’s anti-*Plasmodium* defense, shedding light on *Anopheles*-*Wolbachia*-*Plasmodium* interactions and their potential for *Wolbachia*-based malaria control.

## 4 Rationale for integration

The rationale is rooted in both the complexity of the disease and the challenges associated with existing methods of control. Malaria, primarily spread by mosquito vectors, is influenced by a multitude of factors, ranging from climatic conditions and human behavior to the biological aspects of the mosquito itself. This complexity means that no single intervention is sufficient to control or eliminate the disease comprehensively. Traditional methods of malaria control, such as the use of ITNs, IRS, and chemical repellents, have proven effective but are not without limitations. The increasing incidence of insecticide resistance among mosquito populations is a growing concern, compromising the long-term sustainability of these methods. Moreover, the environmental impacts of chemical-based strategies cannot be ignored, making it crucial to explore eco-friendly alternatives.

Emerging *Wolbachia*-based strategies offer a fresh approach to intervention. *Wolbachia* are symbiotic bacteria that, when introduced into mosquito populations, can either reduce the lifespan of mosquitoes or inhibit their ability to transmit the malaria parasite. These methods are promising for several reasons. They are environmentally friendly and sustainable, posing little risk of developing resistance. They also target the mosquito’s biology, thus providing a novel mechanism of action that can complement traditional methods.

However, the implementation of *Wolbachia*-based strategies is not without its challenges. Using *Wolbachia*-infected mosquitoes to combat malaria requires a stable relationship between them. This involves understanding how *Wolbachia* interacts with the mosquito’s immune system, as it can evade antimicrobial defenses. Additionally, the mosquito’s natural microbiota and antibiotics play a role in ensuring stable infections. Additionally, different *Wolbachia* strains can enhance malaria parasite infection (Hughes et al., 2012; Hughes et al., 2014b; Murdock et al., 2014). Overall, this approach holds promise but requires careful consideration and ongoing research. Factors like the cost of large-scale releases, community acceptance, and potential ecological impacts necessitate careful planning and monitoring. This is where the merit of an integrated approach comes in. By combining *Wolbachia*-based strategies with traditional methods, there is a potential to improve the effectiveness of interventions while mitigating the drawbacks associated with each approach individually.

Several case studies and pilot projects have successfully demonstrated the value of an integrated approach. These projects have combined *Wolbachia*-infected mosquitoes with existing vector control strategies, resulting in enhanced control of disease transmission. Such integrated methods could offer a more nuanced and adaptive strategy, capable of being tailored to specific local conditions. This integration allows for a multipronged attack on the disease, drawing on the strengths of both traditional and emerging methods while minimizing their respective weaknesses. Given the severe global impact of malaria, particularly in impoverished regions, exploring and implementing integrated strategies should be a priority for both research and public health communities.

## 5 Case studies and pilot projects

*Wolbachia*-infected mosquitoes, successful in Cairns, Australia in, 2011 (Hoffmann et al., 2011), are now being used in over 14 countries for population replacement programs (World Mosquito Program, 2017). These initiatives release *Wolbachia*-infected mosquitoes on purpose to manage mosquito populations. Eliminating dengue with *Wolbachia*-infected mosquitoes was also applied in Brazil because after not seeing cases for over 20 years, dengue came back to Brazil in, 1981. A large-scale release program of *Wolbachia*-infected mosquitoes (*w*Mel strain) in Rio de Janeiro, Brazil, aimed at reducing the incidence of dengue and chikungunya. Despite challenges in Rio de Janeiro, releasing the *w*Mel strain resulted in a significant 38% reduction in dengue and a 10% reduction in chikungunya, offering insights for future mosquito release programs (Dos Santos et al., 2022). In Yogyakarta, Indonesia, a study by Utarini, Indriani (Utarini et al., 2021) revealed a notable 77% decrease in dengue transmission in areas with released *Wolbachia*-infected mosquitoes, as shown in randomized controlled trials. Indriani, Tanamas (Indriani et al., 2023) analysed that releasing *Wolbachia*-infected mosquitoes in Yogyakarta, Indonesia, between January, 2006 and May, 2022 significantly lowered the number of severe dengue cases by 83% in fully treated areas. Insecticide spraying also decreased by 83% in treated areas, indicating that *Wolbachia* intervention effectively reduced both severe dengue cases and the need for spraying in routine health efforts.

The *w*AlbB-infected *Ae. aegypti* released in the USA, Myanmar, Malaysia, and China led to reduced human dengue incidence (Laven, 1967; Nazni et al., 2019; Zheng et al., 2019; Crawford et al., 2020). Additionally, the *w*AlbB strain was also released in Singapur in, 2018 and resulted in a 71-88% deduction in dengue incidence (Project Wolbachia–Singapore Consortium & Ching, 2021). Tantowijoyo, Tanamas (Tantowijoyo et al., 2022) confirmed that introducing *Wolbachia* to reduce dengue had effective outcomes, as the mosquito population and insecticide resistance showed no significant differences between treated and untreated groups in the Applying *Wolbachia* to Eliminate Dengue trial. Turner, Quyen (Turner et al., 2023) proposed that *Wolbachia*-infected mosquitoes to control dengue in high-risk urban areas of Vietnam is cost-effective, with a projected cost of $420 per health year saved. It was proposed that Releasing *Wolbachia* mosquitoes in ten high-burden cities that make up 40% of the country’s dengue cases, could prevent 6.2 million cases over 20 years, bringing significant health and economic benefits. Population suppression and lower dengue transmission are confirmed by all these field releases but the duration of suppression remains unclear due to limited post-release monitoring in studies.

Additionally, advancements in the field of paratransgenic research, specifically focusing on Anophelines, have opened new doors for combating malaria. The groundbreaking study conducted by (Mancini et al., 2016) serves as a case in point. In their research, they found that primary malaria vectors, such as *An. stephensi* and *An. gambiae* were not only capable of disseminating genetically modified bacteria but did so at an effective rate. This discovery has significant implications for future malaria control efforts. By manipulating bacteria within the mosquito gut, there is the potential to reduce or even block the transmission of the malaria parasite. Moreover, the study demonstrated trans-stadial diffusion pathways in *An. gambiae*, indicating the bacteria’s persistence across different life stages of the mosquito.

Recent mathematical modeling work by (Matsufuji and Seirin-Lee, 2023) has contributed to our understanding of how IIT could be employed for disease control. Their model offers valuable insights into effective release strategies and cost considerations, particularly in terms of the impact on human infection rates. In Haiti, a distinct mathematical model was crafted to evaluate *Wolbachia*-based interventions specifically (Andreychuk and Yakob, 2022). This model considers various stages of the mosquito life cycle, the maternal transmission of *Wolbachia*, and the effects of CI. Notably, the model indicates that releasing *Wolbachia*-infected mosquitoes during the dry season is more effective than during the wet season, offering a critical guideline for field implementations. These case studies and mathematical models collectively enhance our comprehension of how traditional and *Wolbachia*-based strategies can be successfully integrated. They not only validate the potential of such integrated approaches but also offer practical guidelines and insights that can inform large-scale deployments. Therefore, they serve as exemplary models that could guide the future scaling of these integrated strategies to combat malaria effectively.

### 5.1 Risk analysis of *Wolbachia*-release

To test *Wolbachia*-infected mosquitoes in Australia, the Commonwealth Scientific and Industrial Research Organisation (CSIRO) was asked to check if it could be risky. The risk analysis focused on the possibility that the release might cause more harm than naturally occurring *Ae. aegypti* over the next 30 years. The risk analysis had five stages. First, experts were asked about potential problems with releasing mosquitoes in a workshop and they identified 50 issues. The second, a Cairns workshop, concluded that reducing *Ae. aegypti* wouldn’t harm ecosystems. In the third stage, a workshop in Cairns involving both experts and community members used a tool called Bayesian Belief Nets (BBNs) to understand the relationships between different issues. Although they didn’t completely agree, they got some initial ideas. Email solicitation in the fourth stage highlighted high uncertainty and the final Brisbane workshop aimed to address uncertainties and achieve consensus priors for risk calculation. The final BBNs estimate a 12.5% chance of experiencing some harms labeled as ‘Cause More Harm’ within 30 years of the release, potentially from various hazards. When assessing risk using expert scores, there was no indication of high-risk hazards. For more details see (Murphy et al., 2010).

At that time before field testing of *Wolbachia*-infected mosquitoes, an extensive social research and community engagement initiative was also conducted in Cairns, Northern Australia by Popovici, Moreira (Popovici et al., 2010). The predominant concern among surveyed community members and stakeholders was ensuring the safety of the proposed approach for humans, animals, and the environment. The first concern was whether *Wolbachia* could be transferred to humans. And no evidence suggests that *Wolbachia*, found in mosquitoes, can transfer to or harm humans. Studies since the, 1930s confirm its non-pathogenic nature (Hertig, 1936), and human exposure for thousands of years has shown no adverse effects. Human volunteers in the project received thousands of bites without harm, indicating no risk of *Wolbachia* transfer or harm to humans. PCR analysis of mosquito saliva confirms *Wolbachia’s* absence (Moreira et al., 2009b). Despite years of exposure to *Wolbachia*-infected mosquitoes, antibody response studies in volunteers have revealed no specific IgG antibodies against *Wolbachia*. These findings address concerns about *Wolbachia* negatively affecting the environment, emphasizing a negligible risk associated with long-term exposure to these mosquitoes. *Wolbachia*, transmitted vertically, degrades naturally, posing the minimal risk of horizontal transfer. Laboratory challenges in cross-species transmission and natural experiments show rare horizontal transmission, affirming low environmental risk. Additionally, experiments conducted in a semi-field facility with thousands of *Wolbachia*-infected mosquitoes bred for months showed no transmission to environmental samples (soil, plants, and arthropods). The results suggest that horizontal transmission of *Wolbachia* to non-target species is infrequent, and even if it occurs, the impact is likely negligible, given the widespread occurrence of *Wolbachia* in nature without negative effects on infected insect populations.

Besides all the above-mentioned analyses, the Eliminate Dengue Program has undergone several government risk assessments and received regulatory approval in five countries at that time (World Mosquito Program, 2017). Each assessment concluded that releasing mosquitoes infected with *Wolbachia* poses minimal risk, with low chances of *Wolbachia* transferring to other organisms and causing harm even if such a transfer occurs. Although the horizontal transfer of *Wolbachia* between host species is known to occur, it is generally considered rare in science (Hamm et al., 2014). There isn’t any indication of horizontal transfer in natural trials, like the cohabitation of *Ae. aegypti* and *Ae. albopictus* that is *Wolbachia*-infected. No traces of *w*Mel were found while specifically tested for its transfer from *Ae.* aegypti to other species in their natural habitat. Eliminating Dengue’s methods is not likely to introduce this strain to new locations, as the natural host of *w*Mel, *Drosophila melanogaster*, already shares its geographic distribution with *Ae. aegypti*. For the most raised question about bacterium’s genetic makeup changed over time, Dainty, Hawkey (Dainty et al., 2021) wanted to see if this could affect its ability to control dengue. The results showed that the *w*Mel genome remained mostly the same for up to 7 years after its initial release in Cairns, Australia. This suggests that the genetic material of *w*Mel is stable in its new mosquito host, which is good news for its long-term effectiveness in controlling dengue.

## 6 Malaria and Pakistan

Malaria poses a significant threat in flood-affected Pakistan, impacting over 33 million people, damaging, 1543 health facilities, and submerging over a third of the country. Damages exceeded $15 billion, destroying one million homes, flooding 2 million acres of crops, and damaging 500,000 kilometers of roads. The aftermath brought a severe malaria outbreak, the worst since, 1973, with mosquitoes surging before the waters receded (Pakistan responds to surge in cases following the 2022 floods, 2022). The number of malaria cases reported in Pakistan increased fourfold from, 2021 to, 2022 (World Health Organization, 2022c). With 78% of all verified cases in, 2022 coming from Sindh and Balochistan, these two areas were especially severely affected. With limited access to healthcare, insufficient medical supplies, and ongoing outbreaks, including COVID-19, there is a very high risk of severe health consequences. COVID-19 measures increased mosquito exposure, raising disease risk, and disruptions like lockdowns ceased important control efforts (Noreen et al., 2021). Challenges, like doubled malaria cases, a high *P. falciparum* ratio, and shortages in medicines and supplies, hinder effective responses, spreading vector-borne diseases.

Malaria is present year-round, and most people report mosquito bites at night, especially outside their homes. Those who spend more time outdoors face a higher risk of getting infected with malaria. Pakistan was doing well in fighting malaria, especially in crowded places like Punjab, by improving health systems and managing water and rice cultivation. Before the, 2022 monsoon season, Pakistan implemented WHO-recommended strategies to control mosquito-borne diseases and distributed free LLINs to prevent mosquito bites. The government offered free diagnostic tests, chloroquine, and primaquine, while also implementing IRS and larval control supported by global and government funds (Arshad et al., 2022). But then, the flood happened, disrupting efforts to control malaria by affecting hard-to-reach places and putting progress at risk. Pakistan also faces challenges in controlling malaria, primarily due to climate, urbanization, and a lack of public awareness. Climate change is a big problem for Pakistan. In May, 2022, there was a severe heatwave with temperatures reaching 51°C. Pakistan’s expected temperature rise is higher than the global average (Chaudhry, 2017). Due to these climate changes, The impact of malaria is also shaped by factors like poverty with the country projected to have a 37.2% poverty rate in, 2023 World Bank (2023) and restricted healthcare access, ranked 154th among 195 countries in terms of Healthcare access (Institute for Health Metrics and Evaluation, 2023).

Pakistan is expected to experience more extreme weather like cyclones or intense monsoons due to higher sea and atmospheric temperatures (United Nations Development Programme, 2020). Several tools are used for malaria vector control, but current interventions mainly focus on indoor-biting vectors. Preventative measures such as LLINs and IRS, are crucial for reducing transmission. However, the supply of LLINs is insufficient, and their utilization among flood-affected individuals is low. Resources for the IRS are also extremely limited. However, these approaches are losing effectiveness, especially in areas where outdoor-biting vectors sustain malaria transmission. Recent floods make many areas unreachable by healthcare associations and it’s difficult to supply them with LLINs IRS and medications. So, what if we use something that can reach these areas by itself? Interestingly mosquitoes are the best vectors to carry anything whether it’s something dangerous like *Plasmodium* or something beneficial like *Wolbachia*. Advances in genetic engineering and gene-drive modeling provide new ways to target malaria vectors with rapidly changing biting behavior and using *Wolbachia* infection to prevent the spread of *Plasmodium* in mosquito populations is a very promising idea (Shaw et al., 2016).

## 7 Ethical considerations

Traditional methods of malaria control such as IRS, ITNs, and chemical repellents have been widely used and are effective to varying extents. However, insecticides are a key component, and their use raises several ethical questions. First among them is the issue of environmental impact. The use of chemical insecticides can be harmful to non-target organisms, disrupting local ecosystems. It is crucial that these environmental effects are assessed before large-scale implementation, and that safer, more eco-friendly alternatives are pursued where possible. Another important consideration is community consent and education. It’s essential that the community understands the benefits and risks associated with these interventions, and that informed consent is obtained before implementation. Affected communities should be included in decision-making processes, and their concerns should be addressed transparently and respectfully.

In addition, equitable access to these control measures is a significant ethical concern. In regions where malaria is endemic, impoverished communities are often the most affected. Programs should be designed and implemented in a manner that ensures equal access to interventions like IRS and ITNs, irrespective of socioeconomic status. The occupational health of those who are applying insecticides in IRS or producing ITNs should also be taken into account. Proper safety guidelines should be established, and workers should be adequately trained and equipped with protective gear. Regarding chemical repellents, there are also concerns about long-term health effects, especially for children and pregnant women. Clear guidelines about the safe use of these products need to be communicated, and alternatives should be provided for those who are at higher risk.

The use of *Wolbachia*-based methods in controlling malaria has garnered considerable attention due to its environmentally friendly nature and general ethical acceptability (Mbabazi et al., 2021). Nevertheless, questions surrounding long-term ecological impacts persist, along with ethical concerns about the authority to make decisions affecting entire ecosystems. This underscores the importance of extensive public engagement and the development of robust ethical frameworks to govern the application of this biotechnology. To enrich these points, public consultations and community engagement should be mandatory before the deployment of *Wolbachia*-based interventions. Such engagement not only serves to educate the public but also provides a platform for community members to voice concerns or suggestions, thus making the project more participatory and socially responsible.

Moreover, comprehensive ethical guidelines should be developed, scrutinized, and implemented. These guidelines must take into account not just the potential health benefits but also any possible unintended ecological consequences. Ethical review boards, comprising experts from diverse backgrounds such as biology, ethics, and community leadership, should oversee the planning, implementation, and post-intervention stages. Furthermore, ongoing surveillance mechanisms should be established to monitor long-term ecological impacts, and this information should be transparently communicated to the public and relevant stakeholders. These mechanisms can serve as a safeguard to promptly identify and mitigate any unforeseen environmental repercussions.

Finally, given the questions about who has the right to make decisions affecting ecosystems, it is crucial to involve not just local but also international governance bodies to ensure a diverse and representative decision-making process. This would include health agencies, environmental organizations, and local communities that could be affected by these interventions. By adopting these measures, the deployment of *Wolbachia*-based interventions can be more ethically sound, environmentally responsible, and socially acceptable, thereby maximizing the potential for this promising approach in the fight against malaria.

## 8 Discussion

Global efforts to combat malaria are seriously disadvantaged by *Anopheles* mosquito insecticide resistance. Despite progress in the last decade, global malaria deaths persist at around 400,000/year, highlighting the ongoing need for prevention and control efforts alongside bed nets and indoor spraying (McGraw et al., 2001). IVM strategies, particularly using the symbionts with emphasis on the *Wolbachia*-based strategies., show promise in reducing mosquito populations for vector-borne disease control. This approach, compared to insecticide-based methods, may offer cost-effectiveness and environmental benefits. *Wolbachia’s* protective mechanism involves immune priming, enhancing the host’s defenses against pathogens (Suh et al., 2023). However, variations in the effects of *Wolbachia* strains, as reported with the *w*AlbB strain, suggest differing interactions with hosts and pathogens, providing insights into molecular processes that hinder pathogen development in mosquitoes (Yu et al., 2022). Studies suggest that *Wolbachia* could offer protection against human malaria if stably introduced into *Anopheles* mosquitoes. Using *w*MelPop-CLA, *Ae. aegypti* mosquitoes saw a 67-88% reduction in *P. gallinaceum*, while *An. gambiae* females with *w*MelPop had 75-84% lower *P. berghei* levels, highlighting *Wolbachia’s* potential to reduce malaria parasites in *Anopheles* mosquitoes (Ashour and Othman, 2020). Research on its ability to stop *Plasmodium* transmission in mosquito larvae is still underway. Mechanisms such as enhanced innate immune responses, host microRNA regulation, and competition for fatty acids are being investigated as potential means of controlling malaria.

Historically, the main malaria vector, *An. gambia*e and *An. funestus*, exhibited indoor feeding patterns, primarily biting indoors during late-night peak activity (Briët and Chitnis, 2013). This behavior aligns with the time when most people are indoors and asleep. There is increasing evidence of malaria vectors altering their biting behaviors, targeting times and locations where people are not adequately protected (Meyers et al., 2016). To address changes in host choice and resting patterns of malaria vectors evading ITNs, consider behavioral research, targeted control, improved net design, monitoring, community education, diverse vector control methods, insecticide resistance management, collaboration, environmental management, and policy support.

### 8.1 Future prospects and challenges

The Global Technical Strategy for Malaria, 2016–2030 (GTS) aims to eliminate malaria in a minimum of ten countries by, 2020, followed by 20 countries by, 2025, and eventually 30 countries by, 2030 (World Health Organization, 2023). Sub-Saharan Africa, which is where 90% of the world’s malaria cases originate, continues to pose the greatest challenge. A few nations in Africa have experienced success, including Senegal, Zanzibar, and a large portion of southern Africa (Roux et al., 2021). It is crucial to assess whether transinfected *Wolbachia* can effectively reduce malaria transmission, given the ongoing debate surrounding *Wolbachia*’s impact on malaria. While *Wolbachia* has demonstrated strong suppression of dengue and other arboviruses when transferred to new hosts, achieving the same level of suppression for malaria remains uncertain. One potential approach is attempting to transfer the newly identified *Anopheles *Wolbachia** strain to *An. stephensi*, as successful transinfections have been achieved in this species. Additionally discovery of native *Wolbachia* infections in *An. gambiae* reducing malaria transmission is significant. However, challenges persist in utilizing *Wolbachia* for malaria control, particularly uncertainty regarding its ability to induce CI in *Anopheles*. Environmental factors and low *Wolbachia* levels may affect CI expression, while higher levels could enhance *Plasmodium* protection, necessary for effective control strategies. However, implementing a disease suppression program by replacing natural populations with *Wolbachia*-carrying mosquitoes presents numerous challenges, including potential negative effects on host fitness that could hinder invasion, as seen with the *w*MelPop infection in *Ae*. *aegypti* (Ogunlade et al., 2021). Nonetheless, recent research suggests that *Anopheles* disease vectors can host *Wolbachia* infections, opening up new avenues for their use in disease suppression.

Incompatible Insect Technology (IIT) using *Wolbachia*-infected male mosquitoes effectively reduces the fecundity and fertility of wild mosquito populations. This strategy shows promise in eliminating invasive mosquitoes, like *Ae. aegypti* and *Ae. albopictus*, and decreasing the incidence of dengue, chikungunya, and Zika (World Health Organization, 2022a) so this strategy may be applied to control malaria. Releasing *Wolbachia* mosquitoes doesn’t pause the epidemic immediately but leads to a gradual mosquito population decline over months. The issue here is that using *Wolbachia* to control vectors like *Ae. aegypti* and *Anopheles* species faces challenges as these main vectors are not naturally infected. Laboratory transinfection is needed for stable transmission, and culturing the bacteria, which rely on insect cells, is challenging due to the slow and complex process. In countries such as Pakistan, where *Wolbachia* release initiatives have not been implemented, the establishment of policies and the expansion of such projects on a large scale pose significant challenges. Initiating projects of this nature in Pakistan would require thorough community education to dispel misconceptions, particularly regarding the fact that the bite of a *Wolbachia*-infected mosquito is harmless and does not involve the transfer of pathogens. Successful implementation of pilot projects aimed at controlling vector-borne diseases like malaria necessitates extensive community counseling, followed by active engagement with policymakers.

### 8.2 Recommendations

New bed nets using a mix of pyrethroids and synergists like piperonyl butoxide offer improved prevention but at a higher cost. Concurrently, the development of new non-pyrethroid insecticides is also in progress, though this adds to program expenses. The prospect of utilizing mosquitoes infected with *Wolbachia*, which are incapable of transmitting malaria, offers a promising approach for future advancements in malaria control. Therefore, governments and health agencies should allocate funds for researching *Wolbachia*’s efficacy in malaria control. Initiating pilot projects in various ecological settings could provide invaluable insights into this approach. Developing ethical guidelines in dialogue with affected communities is another imperative step for responsible intervention.

Effective IVM programs involve various elements such as advocacy, collaboration, an integrated strategy, and enhanced human resource capacity. The cornerstone of successful IVM is local decision-making and community participation, which are vital for the sustainability of these efforts. Some countries are still in the planning phase of their IVM strategies, while others, Namibia (Chanda et al., 2015), Swaziland (Cohen et al., 2013), Botswana (Chihanga et al., 2016), Zambia and Zimbabwe (Chanda et al., 2012), have incorporated IVM into their national policies with varying degrees of success.

There is a need to develop the technical capacity and infrastructure for entomological surveillance, which is critical for effective vector control in the fight against diseases like malaria. Targeted training of entomological technicians in countries like Burundi, Eritrea, Guinea, and Zambia, resulted in significant reductions in malaria burdens, sometimes up to 99% (Chanda et al., 2015) can serve as an instructive example for Pakistan. Pakistan may significantly reduce the incidence of malaria by investing in entomological expertise and adjusting strategies to local conditions. Insecticide resistance poses a potential limitation to IVM programs, which rely heavily on insecticides like long-lasting nets and indoor spraying. To enhance vector control efforts effectively, it’s crucial to combine innovative strategies like genetically modified mosquitoes, *Wolbachia*-based mosquitoes, diverse mosquito traps, spatial repellents, and entomopathogenic fungus-impregnated targets with conventional insecticides.

In sum, combating malaria is a multifaceted problem that requires an integrated approach, combining pharmacological, biological, and genetic strategies. Drug resistance is a significant hurdle, and while vaccines offer a ray of hope, they have their limitations. Biological strategies such as *Wolbachia*-based control offer promising but complex solutions. In the end, the fight against malaria will likely require a combination of these strategies, carefully tailored to the ecological and epidemiological specifics of different regions.

## 9 Conclusion

The battle against malaria is an ongoing struggle that demands innovative and multi-dimensional solutions. While traditional methods of control, such as insecticide-treated bed nets and indoor residual spraying, continue to play a critical role, their effectiveness is increasingly compromised by challenges like insecticide resistance. The integration of emerging *Wolbachia*-based interventions offers a new channel of promise. It shows promise in controlling dengue transmission, but its application for malaria control is limited due to the lack of infection in key vectors. Identifying new strains may allow for novel interventions in anopheline mosquitoes. Concerns about pathogen enhancement need consideration, and the long-term benefits of *Wolbachia* will require extensive disease surveillance and large-scale studies. While multiple malaria prevention methods effectively reduce incidence and prevalence, some studies show mixed results. More evidence is needed for individual and combined malaria prevention methods to inform future research and strategies in endemic countries. Only through such an integrated approach can we hope to make significant strides in reducing the global burden of malaria.

## Author contributions

IM: Conceptualization, Investigation, Writing – original draft. AC: Writing – review & editing. SS: Writing – review & editing. MA: Writing – review & editing. MS: Conceptualization, Supervision, Writing – review & editing.
